# Marker-assisted backcrossing: a useful method for rice improvement

**DOI:** 10.1080/13102818.2014.995920

**Published:** 2015-02-26

**Authors:** Muhammad Mahmudul Hasan, Mohd Y. Rafii, Mohd R. Ismail, Maziah Mahmood, Harun A. Rahim, Md. Amirul Alam, Sadegh Ashkani, Md. Abdul Malek, Mohammad Abdul Latif

**Affiliations:** ^a^ Department of Crop Science, Faculty of Agriculture, Universiti Putra Malaysia, 43400 UPM Serdang, Selangor, Malaysia; ^b^ Laboratory of Food Crops, Institute of Tropical Agriculture, Universiti Putra Malaysia, 43400 UPM Serdang, Selangor, Malaysia; ^c^ Deparment of Biochemistry, Faculty of Biotechnology and Biomolecular Sciences, Universiti Putra Malaysia, 43400 UPM Serdang, Selangor, Malaysia; ^d^ Agrotechnology and Bioscience Division, Malaysian Nuclear Agency, Bangi, 43000 Kajang, Selangor, Malaysia; ^e^ Department of Agronomy and Plant Breeding, Shahr-e-Rey Branch, Islamic Azad University, Tehran, Iran; ^f^ Department of Plant Breeding, Bangladesh Institute of Nuclear Agriculture, Mymensingh, Bangladesh; ^g^ Department of Plant Pathology, Bangladesh Rice Research Institute, Gazipur, Bangladesh

**Keywords:** conventional breeding, gene introgression, markers, MAB, rice improvement

## Abstract

The world's population is increasing very rapidly, reducing the cultivable land of rice, decreasing table water, emerging new diseases and pests, and the climate changes are major issues that must be addressed to researchers to develop sustainable crop varieties with resistance to biotic and abiotic stresses. However, recent scientific discoveries and advances particularly in genetics, genomics and crop physiology have opened up new opportunities to reduce the impact of these stresses which would have been difficult if not impossible as recently as the turn of the century. Marker assisted backcrossing (MABC) is one of the most promising approaches is the use of molecular markers to identify and select genes controlling resistance to those factors. Regarding this, MABC can contribute to develop resistant or high-yielding or quality rice varieties by incorporating a gene of interest into an elite variety which is already well adapted by the farmers. MABC is newly developed efficient tool by which using large population sizes (400 or more plants) for the backcross F_1_ generations, it is possible to recover the recurrent parent genotype using only two or three backcrosses. So far, many high yielding, biotic and abiotic stresses tolerance, quality and fragrance rice varieties have been developed in rice growing countries through MABC within the shortest timeframe. Nowadays, MABC is being used widely in plant breeding programmes to develop new variety/lines especially in rice. This paper reviews recent literature on some examples of variety/ line development using MABC strategy.

## Introduction

Recurrent backcrossing is a traditional breeding method commonly employed to transfer alleles at one or more loci from a donor to an elite variety.[1,2] The expected recurrent parent (RP) genome recovery would be 99.2% by six backcrosses, which is most similar to improved variety. The proportion of the RP genome is recovered at a rate of 1−(1/2) ^t+1^ for each of the generations of backcrossing.[3] However, any specific backcross progeny (BC_3_ or BC_2_), they will be deviated during crossing over resulting in a great chance to get the expected result that is not possible to detect phenotypically. For example, in BC_1_ population, theoretically the average percentage of the RP genome is 75% for the entire population. But some individuals possess more or less of the RP genome than others. Those individuals that contain the highest RP genome are selected. Advancement of genomic research in rice and completion of the rice genome sequence have been developed and it has been possible to detect and map finely a number of genes through linkage to DNA markers. Backcrossing is a widely used technique in rice breeding for introgression or substitution of a target gene from donor parent to recipient. It provides a precise way to improve varieties that excel in a large number of attributes.[1,4,5] The main purpose of backcrossing is to decline the donor genome content into the progenies.[6] Backcross breeding has been adopted in the South and Southeast Asia [7,8] as breeding strategy to improve elite varieties such as KDML105, Basmati and Manawthukha for their resistances to blast.[9] In the meantime, a potential backcrossing approach has been established by applying molecular markers named as simple sequence repeats (SSRs) and single nucleotide polymorphisms. One of the early benefits of molecular markers in plants is to enhance the backcrossing process by reducing the number of backcrosses required to recover the recurrent phenotype.[10] This approach was first reported for rice by Chen et al. [11] They introduced resistance to bacterial blight (BB) disease into Chinese hybrid parents. It was also described for submergence tolerance using the *sub1* gene at International Rice Research Institute (IRRI).[12]

Therefore, molecular markers are the tools that can be used to detect the presence of desire character which is economically important. Marker assisted backcrossing (MABC) is an attractive tools for breeding vastly using in a large number of research aim at identifying genomic regions of interest. Molecular markers that are tightly linked with economically important traits have been identified and/or used for MABC in rice including resistance of BB, blast, brown plant hopper, green leaf hopper, gall midge, virus infections, and tolerance to salinity, submergence, drought, cold, semi-dwarf, grain quality and much more. As for molecular marker based research no need of genetic transformation and cultivars are developed by MABC, therefore, there is no contradiction or ethical issues as already raised with transgenic crops. This review focuses on the potential application of MABC for the improvement of rice. This updated information will be a helpful guidance for rice breeders to develop a durable biotic and abiotic stress tolerant rice variety.

## The backcross approach

The backcrossing approach was proposed by Harlan and Pope.[13] Since then, backcrossing has become a widely used plant breeding approach in diverse crop species.[5,14,15] This method is most commonly used to incorporate one or a few traits into an adapted or elite variety. In most cases, the elite variety used for backcrossing has a large number of desirable attributes but is deficient in only a few characteristics.[1] The other parent, called the ‘donor parent’, possesses one or more genes controlling an important trait which is lacking in the elite variety. The prime aim of backcross breeding is to transfer one or more genes of interest trait from donor parent into the background of the improved variety and recover the RP genome by eliminating the undesirable genes (linkage drag) as quick as possible. Those unwanted genes transferred through linkage drag cause a negative effect on agronomical traits, such as low yield or disease susceptible. Depending on the linkage distances, the size of the flanking regions can be decreased by additional backcrossing [16] although breeders have not had any direct control over the size of the region or the recombination breakpoints.

**Figure 1.**
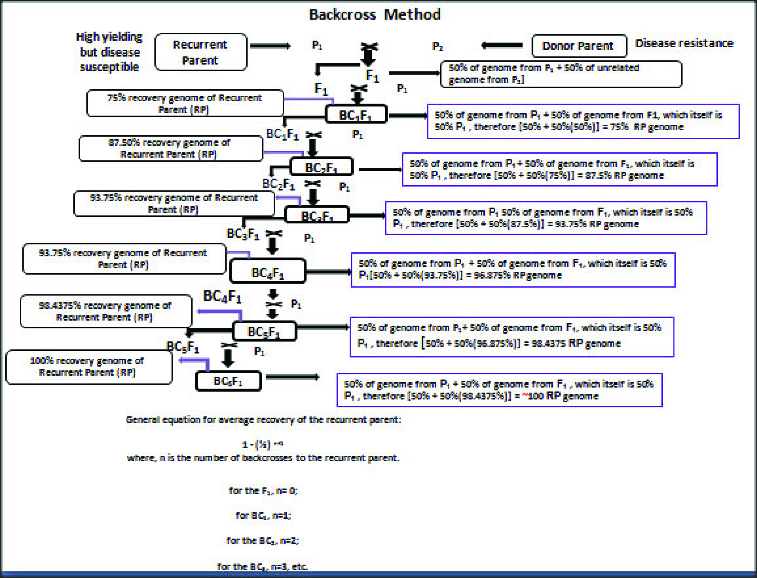
Schematic representation of conventional backcrossing (Modified from George Acquaah [17]).

Backcross populations are made by crossing the RP with the donor parent to produce an F_1_ hybrid and then crossing the F_1_ with the RP to produce the first backcross generation (BC_1_F_1_ or just BC_1_). After phenotypic screening, the next backcross generation is made by crossing selected BC_1_ plants (that have been screened for the target trait) with the RP to produce the BC_2_. Subsequent backcross populations are made by repeatedly crossing the selected backcross (BC) plants with the RP. It should be emphasized that backcross progeny with the target trait must be selected based on phenotype during each round of backcrossing. There is no absolute number for how many backcrosses need to be performed but generally between six to eight backcross generations are performed. After the final backcross generation, selected individuals are self-pollinated so that selected lines will be homozygous for the target trait. Typical backcross breeding is shown in Figure 1. The recessive backcross scheme can also be used for target traits but is difficult to get desired result accurately due to phenotype based on single plants. The end product of a backcrossing programme is to obtain lines that are as similar as possible to the RP but also possess the target traits.

### Essential elements for successful backcross programme

Three main factors are essential to the success of a backcross breeding programme: the selection of the RP, an effective means to screen for the target trait(s) and the number of backcrosses used to reconstitute the RP.[1,18]

#### Recurrent parent

The choice of the RP must be carefully made based on the factors such as agronomic performance, traits, target environment and popularity with farmers.

#### Screening for target trait

There must be an effective method for phenotypic screening (i.e. a method which clearly discriminates between segregating progeny) due to the number of rounds of crossing. Traits with high heritability will be more effectively incorporated compared to traits with low heritability.

#### Number of backcrosses

In the early backcross generations, breeders may visually select which progeny most closely resembles the RP. However at later generations (i.e. after BC_2_), it may be impossible to discriminate between backcross progeny and the RP based on individual plants. In order to retain the desirable characteristics of the RPs, additional backcrosses must be made on the assumption that additional backcrossing until at least BC_6_ will restore the RP as much as possible (Figure 1).

### Marker-assisted backcrossing (MABC)

MABC is a precise and an effective method to introgress a single locus controlling a trait of interest while retaining the essential characteristics of the RP.[19] It is known that MABC is effective for genes or quantitative trait loci (QTLs) with large variations in phenotype. MABC is the process of using markers to select for target loci, minimize the length of the donor segment containing a target locus and/or accelerate the recovery of the RP genome during backcrossing.[20,21] The main objective of MABC is to integrate a targeted gene from agronomical substandard sources (the donor parent) into an exclusive breeding line (the RP). The anticipated product is an improved line comprising solely the targeted gene from the donor parent, through the recipient parent line existing all over else in the genome. One of the early benefits envisioned with the use of molecular markers in plants was their use to accelerate the backcrossing process by reducing the number of backcrosses required to recover the RP phenotype.[10]

MABC is superior to conventional backcrossing in precision and efficiency. Background selection can greatly accelerate the backcrossing programme compared to using conventional backcrossing.[22] This approach has been widely used and due to the prevalence of several rice ‘mega varieties’ it is likely to continue being a successful approach.[23] MABC involves successive backcrossing to remove the genetic background of the donor while recovering genetic properties of RPs as much as possible. Statistical methods and schedule of backcrosses to create effective MABC have been reviewed in various published papers.[20,21,24] MABC with marker-based genome scanning has allowed a speedy recovery of most recurrent genome in a few crosses.[25,26] MABC can also be used to develop near isogenic lines (ILs) by minimizing carried-over donor segments flanking the target locus, providing precise introgression of individual genes for detailed characterization of the QTLs. Several applications of marker-assisted backcross breeding have been shown in Table 1.

**Table 1.** Examples of marker-assisted backcross breeding in rice.

Sl number	Target trait	Gene (s)/QTL (s)	Type/name of marker(s) used	References	Remarks
1	Bacterial blight (BB) resistance	*Xa21* and *Xa13*	SSR markers	[27]	MAS applied for marker-assisted backcross breeding (Target variety: Taraori Basmati and Basmati 386)
2		*Xa4, xa5, xa13* and *Xa21*	SSR markers	[28]	MAS applied for marker-assisted backcross breeding (Target variety: PRR78 and KMR3)
3		Xa21	SSR markers	[29]	MAS applied for marker-assisted backcross breeding (Target variety: KDML105)
4		*Xa7* and *Xa21*	SSR markers	[30]	MAS applied for marker-assisted backcross breeding (Target line: Yihui1577)
5		*Xa7, Xa21, Xa22* and *Xa23*	SSR markers	[31]	MAS applied for marker-assisted backcross breeding (Target line: hybrid rice restorer line Huahui 1035)
6		*Xa21*	STS (pTA248)	[11]	MAS applied for marker-assisted backcross breeding
7		*Xa21*	STS (pTA248)	[32]	MAS applied for marker-assisted backcross breeding
8		*xa5, xa13* and *Xa21*	CAPS for *xa5*(RG556+DraI) CAPS for *xa13*(RG136+HinfI) STS for *Xa21*(pTA248)	[33]	MAS applied for marker-assisted backcross breeding (Target variety: PR106)
9		*xa5, xa13* and *Xa21*	CAPS for *xa5*(RG556+DraI) CAPS for *xa13*(RG136+HinfI) STS for *Xa21*(pTA248)	[34]	MAS applied for marker-assisted backcross breeding
10		*xa5*	CAPS (RG556+DraI)	[35]	MAS applied for marker-assisted backcross breeding
11		*xa13, xa21*	STS and SSR	[7]	MAS applied for marker-assisted backcross breeding
12		*xa5, xa13* and *Xa21*	CAPS for *xa5*(RG556+DraI) CAPS for *xa13* (RG136+HinfI) STS for *Xa21*(pTA248	[36]	MAS applied for backcross breeding (Target variety: Samba Mahsuri)
13		*xa5* and *xa13*	CAPS for *xa13*(RG136+HinfI) STS for *Xa21*(pTA248)	[37]	MAS applied for backcross breeding (Target variety: Triguna)
14		*xa13 and Xa21*	CAPS for *xa13*(RG136+HinfI) STS for *Xa21*(pTA248)	[7,38]	MAS applied for backcross breeding (Target variety: Pusa Basmati 1)
15	Blast resistance	*Pi-genes, Xa5*	SSR markers	[39]	MAS applied for marker-assisted backcross breeding
16		*Pi5* and *Pi54*	SSR markers	[40]	MAS applied for backcross breeding (Target variety: PRR78, Basmati rice)
17		*Pi1* and *Pi2*	SSR markers	[41]	MAS applied for backcross breeding (Target variety: Ronfeng B hybrid rice)
18		*Pi1*	SSR and ISSR markers	[42]	MAS applied for backcross breeding (Target variety: Zhenshan 97A)
19	Submergence tolerance	*sub*1	SSR markers	[43]	MAS applied for backcross breeding (Target variety: AS996)
20		*Sub1*	SSR markers	[44]	MAS applied for backcross breeding (Target variety: OM1490)
21		*Sub1*QTL	SSR and STS	[8]	MAS applied for backcross breeding (Target variety: Swarna and Samba Mahsuri)
22		*Sub1*QTL	Phenotypic and SSR	[45]	MAS applied for backcross breeding (Target variety: Swarna and Samba Mahsuri)
23		*Sub1*QTL	SSR	[12]	MAS applied for backcross breeding (Target variety: Swarna and Samba Mahsuri)
24	Salt tolerance	*Saltol*	SSR markers	[46]	MAS applied for backcross breeding (Target variety: Q5DB)
25		*Saltol* QTL	SSR markers	[47]	MAS applied for backcross breeding (Target variety: ASS996)
26		*Saltol* QTL	SSR markers	[48]	MAS applied for backcross breeding (Target variety: Bacthom 7)
27	Drought tolerance	QTL	SSR markers	[49]	MAS applied for backcross breeding (Target variety: KMDL105)
28	Phosphorous tolerance	*Pup1*	SSR markers	[50]	MAS applied for backcross breeding
29	Brown Plant hopper resistance	*Bph14* and *Bph15*	InDel marker B14 and B15	[51]	MAS applied for backcross breeding (Target variety: Shengdao 15, Shengdao 16 and Xudao 3)
30		*Bph18*	SSR markers	[52]	MAS applied for backcross breeding(Target variety: Junambyeo)
31	Gall midge resistance	*Gm8*	SSR markers	[53]	MAS applied for backcross breeding
32	Rice stripe resistance	*Stv-bi*	SSR markers	[51]	MAS applied for backcross breeding (Target variety: Shengdao 15, Shengdao 16 and Xudao 3)
33	Thermo sensitive genic male sterility	*tms2, tgms* and *tms5*	SSR markers	[54]	MAS applied for backcross breeding
34	Deep roots	QTLs on chromosomes 1, 2, 7 and 9	RFLP and SSR markers	[55]	MAS applied for backcross breeding
35	Root traits + Aroma	QTLs on chromosomes 2, 7, 8, 9 and 11	RFLP and SSR markers	[56]	MAS applied for backcross breeding
36	Submergence Tolerance + BPH resistance + Bacterial blight resistance + Blast resistance + quality	*Subchr9 QTL, Xa21, Bph* and blast QTLs, and quality loci	SSR and STS	[8]	MAS applied for backcross breeding
37	Heading date	QTLs for heading date *(Hd1,Hd4, Hd5*, or *Hd6)*	RFLP, STS, SSR, CAPS, dCAPs	[57]	MAS applied for backcross breeding
38	Quality	*Waxy*	RFLP	[58]	MAS applied for backcross breeding
39	Tolerance, disease	*Bph* and blast QTLs, and quality loci	SSR and STS	[8]	MAS applied for backcross breeding

The efficiency of marker-assisted backcrossing depends on a number of factors, including the population size of each backcross generation, distance of markers from the target locus and number of background markers used. Data from Hospital [59] showed faster recovery of the RP genome with marker assisted selection (MAS) compared to conventional backcrossing when foreground and background selections are combined (Table 2). The RP genome is recovered more slowly on the chromosome carrying the target locus than on other chromosomes because of the difficulty in breaking linkage with the target donor allele. Methods for optimizing sample sizes and selection strategies in marker-assisted selection have been discussed by several researchers [22,25,60,61] (Table 2).

**Table 2.** Expected recovery of recurrent parent genome comparing conventional and marker assisted backcrossing in subsequent generations

		% recurrent parent genome	
Backcross generation	Number of individuals	Marker-assisted backcross	Conventional backcross
BC_1_	70	79.0	75.0
BC_2_	100	92.2	87.5
BC_3_	150	98.0	93.7
BC_4_	300	99.0	96.9

Source: Hospital [59]

The use of this powerful approach for MABC was first reported for rice by Chen et al. [11] introducing resistance to BB disease into Chinese hybrid parents. It was also described for submergence tolerance using the *Sub1* gene at IRRI.[12] The basic approach has been described in more detail in several papers by this group.[12,19,45] Using large population sizes (400 or more plants) for the backcross F_1_ generations, it is possible to recover the RP genotype using only two or three backcrosses. The three selection steps [19] are as follows.

#### Foreground selection

**Figure 2.**
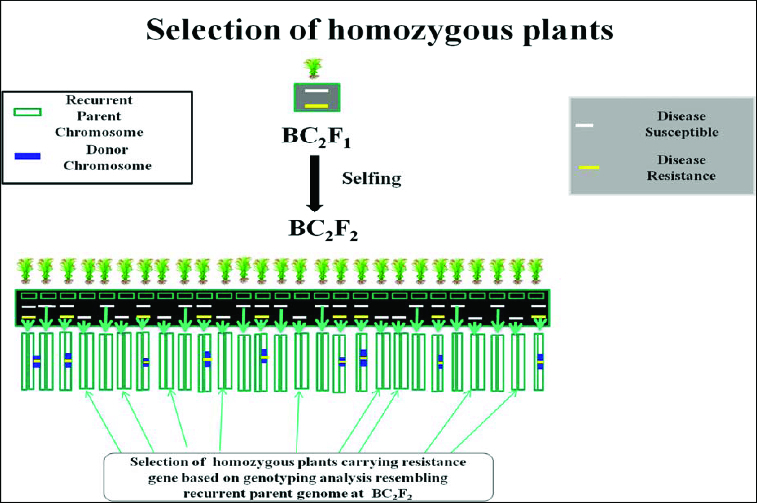
Schematic representation of selection of homozygous plants for the donor allele. Source: modified from IRRI, (2014) with permission (www.knowledgebank.irri.org).

Foreground selection, in which the breeder selects plants having the marker allele of the donor parent at the target locus. The objective is to maintain the target locus in a heterozygous state (one donor allele and one RP allele) until the final backcross is completed. Then, the selected plants are self-pollinated and progeny plants identified that are homozygous for the donor allele (Figure 2). Those markers which have already been developed and they are tightly linked to the target gene or QTL should be used to select the target locus of donor parent in early (BC) progenies for the selection of plants that having the target gene. This is referred to as ‘foreground selection’,[21] although referred to ‘positive selection’.[57] Marker-assisted foreground selection was proposed by Tanksley [62] and investigated in the context of introgression of resistance genes by Melchinger.[63] This may be particularly useful for traits that have laborious or time-consuming phenotypic screening procedures. It can also be used to select for reproductive-stage traits in the seedling stage, allowing the best plants to be identified for backcrossing.

#### Recombinant selection

**Figure 3.**
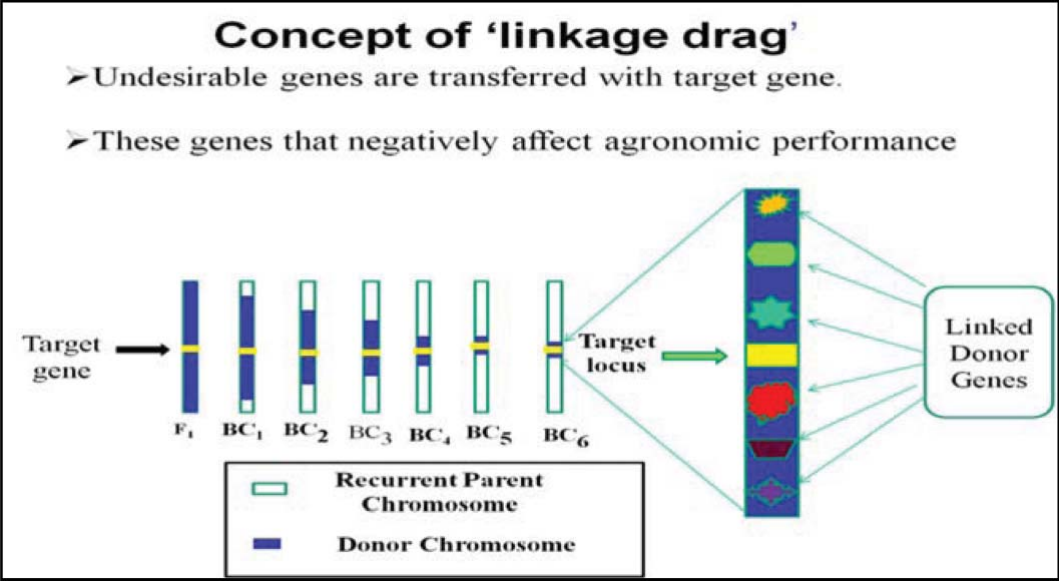
Schematic representation of transferring undesirable genes with target gene. Source: modified from IRRI, (2014) with permission (www.knowledgebank.irri.org).

The second level involves selecting BC progeny with the target gene and recombination events between the target locus and linked flanking markers is termed as ‘recombinant selection’.[19] The purpose of recombinant selection is to reduce the size of the donor chromosome segment containing the target locus (i.e. size of the introgression). This is important because the rate of decrease of this donor fragment is slower than for unlinked regions and many undesirable genes that negatively affect crop performance may be linked to the target gene from the donor parent (i.e. as linkage drag, Figure 3).[15] Using conventional breeding methods, the donor segment can remain very large even with many backcross generations (e.g. >10 cM; [64,65]. By using markers that flank a target gene (e.g. <5 cM) on either side), linkage drag can be minimized. Since double recombination events occurring on both sides of a target locus are extremely rare, recombinant selection is usually performed using at least two BC generations.[25] It must be emphasized that this is only possible for genes or QTLs for which the map position has been well defined. Fine mapping (or high-resolution mapping) is usually required prior to recombinant selection. Furthermore, recombinant selection can minimize the size of the donor chromosome segment, thus reducing ‘linkage drag’ – a ‘universal enemy’ of the plant breeder.[20]

#### Background Selection

Except target locus, all genomic regions can be selected in background selection using RP marker alleles and the selection of target locus is done on the basis of phenotype. This selection is important in order to reduce unnecessary genes (linkage drag, Figure 3) introduced from donor. By using molecular markers, it is easy to delete the unwanted donor alleles in the same genomic region as the target locus.

**Figure 4.**
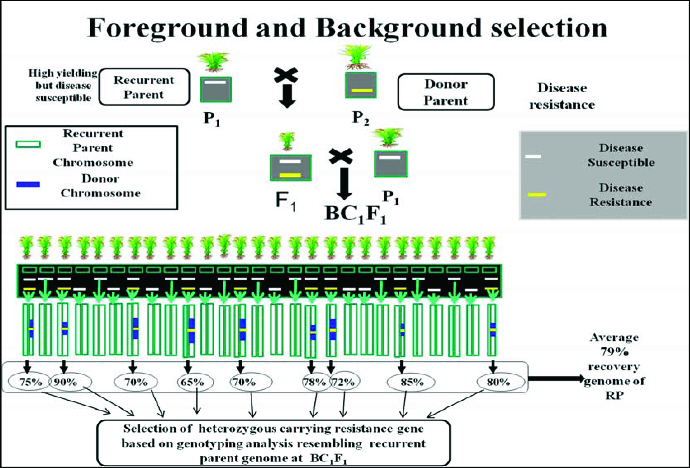
Schematic representation of selection of heterozygous carrying resistance gene based on genotyping analysis resembling RP genome at BC_1_F_1_. Source: modified from IRRI, (2014) with permission (www.knowledgebank.irri.org).

**Figure 5.**
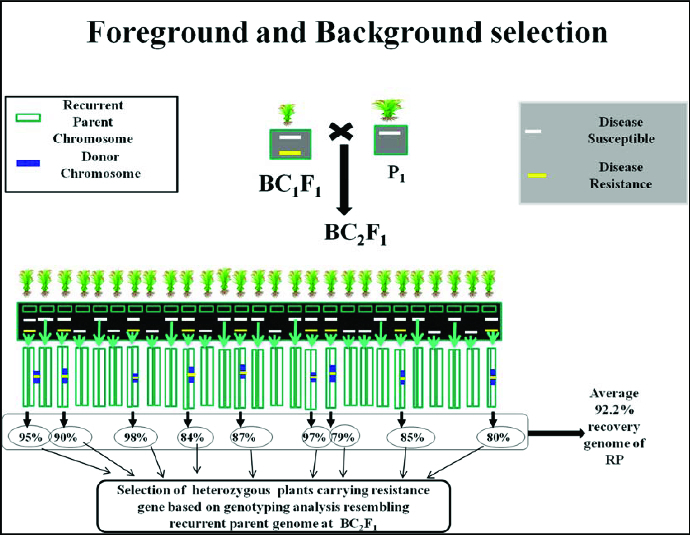
Schematic representation of selection of heterozygous carrying resistance gene based on genotyping analysis resembling RP genome at BC_2_F_1_. Source: modified from IRRI, (2014) with permission (www.knowledgebank.irri.org).

**Figure 6.**
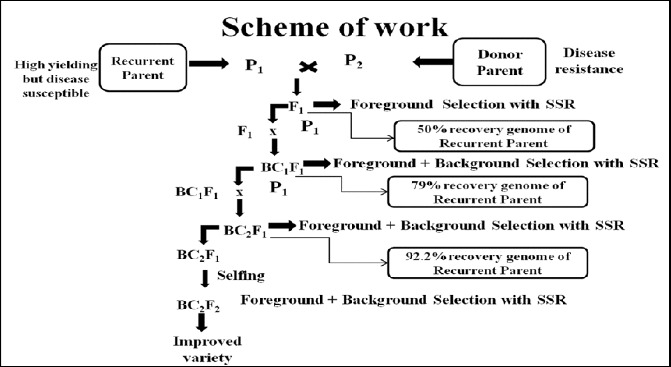
Schematic representation of development of resistant rice variety through marker-assisted backcrossing (MABC). Source: modified from Babu et al. [67].

The third level of MABC involves selecting BC progeny with the greatest proportion of RP genome, using markers that are unlinked to the target locus – we refer to this as ‘background selection’. In the literature, background selection refers to the use of tightly linked flanking markers for recombinant selection and unlinked markers to select RP.[21,25] This was also referred to as ‘negative selection’ by Takeuchi et al. [57] Background markers are markers that are unlinked to the target gene/QTL on all other chromosomes, in other words, markers that can be used to select against the donor genome. This is extremely useful because the RP recovery can be greatly accelerated (Figures 4 and 5). With conventional backcrossing, it takes a minimum of six BC generations to recover the RP and there may still be several donor chromosome fragments unlinked to the target gene. The use of background selection during MABC to accelerate the development of an RP with an additional one or more genes has been referred to as ‘variety development or enhancement’ [45] and ‘complete line conversion’ (Figure 6).[66]

### MABC has several advantages over conventional backcrossing

#### Speed up recovery of recurrent parent genome

**Figure 7.**
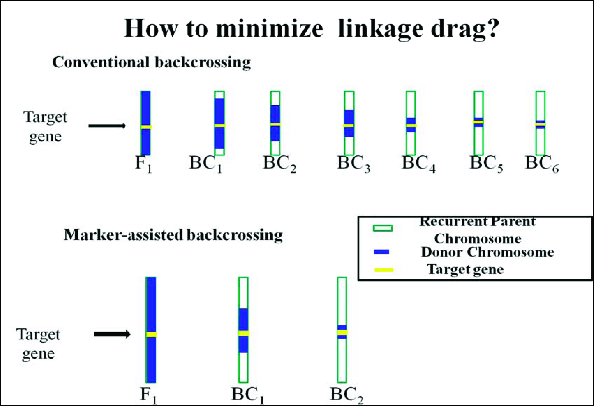
Schematic representation of difference between conventional backcrossing and marker-assisted backcrossing. Source: modified from IRRI, (2014) with permission (www.knowledgebank.irri.org).

Conventional backcross breeding needs six backcrosses to recover the RP genome. But RP genome may be recovered by BC_4_ or BC_3_ even BC_2_ using MABC approach,[21,22,25,68] thus saving two to four BC generations (Figure 7).

#### Consistency

Environmental factors are of great concern and may hamper the expression of plant characteristics**.** But molecular markers are consistent from any significant impact of environmental stresses, which presents great opportunities for selecting molecular markers for MABC.

#### Minimize the linkage drag

To reduce the large number of donor chromosome, minimum six backcross generations are needed whether MABC may need two or three backcross generations. Linkage drag requires many additional backcross generations, and if the undesirable genes are really tightly linked to the target locus it may be difficult to eliminate these genes using conventional backcrossing.[19]

#### Biosafety

Without inoculation of pathogen, the definite characters for disease resistance can be conducted by molecular markers which are tightly linked with the target gene. They also facilitate introgression of genes into high-yielding variety that are disease susceptible**.**

#### Efficiency

By sorting of breeding lines in few generations with the application of molecular markers, it is easy to discard all the progenies from the programme except our targeted lines.

#### More accurate selection

By applying conventional breeding techniques, it is very difficult to identify polygenic characters. But in the case of MABC, it is possible to select on the basis of gene expression using markers.

### Marker-assisted backcrossing in biotic stresses

#### Blast resistance

Recently, Pusa1602 (PRR78+Piz5) and Pusa1603(PRR78+Piz54) lines have been developed through incorporation of blast resistance genes *Piz-5* and *Pi54* derived from donor lines C101A51 and Tetep into the background of PRR78 (highly blast susceptible) through MABC breeding strategy.[40] Foreground selection for the genes *Piz-5* and *Pi5*4 were effected using tightly linked molecular markers, AP5930 and RM206, respectively in two independent backcross series. Background analysis revealed the RP genome recovery up to 89.01% and 87.88% in Pusa1602 and Pusa1603 lines, respectively. The hybrids produced by crossing Pusa6A with improved lines of PRR78, were on par with original Pusa RH10 in terms of yield, grain and cooking quality traits with an added advantage of blast resistance.

Leaf blast resistance line D521, neck blast resistance line D524 and BB resistance have been developed through introgression of leaf resistance gene *Pi1*, neck blast resistance gene *Pi2* derived from donor BL122 and bacterial resistance gene *Xa23* derived from donor CBB23 into an elite, early maturing maintainer line of hybrid rice disease susceptible to both blast and blight, Ronfeng B hybrid rice through marker-assisted backcross breeding programmes.[41] By using three SSR markers (MRG4766, AP22 and RM206), *Pi1*, *Pi2* and *Xa23* have been identified and also used to test the recovery of the genetic background for the improved lines by using 131 polymorphic markers. After four backcrosses, the recovery ratios of the recurrent-parental genome at non-target loci for D521 and D524 were 96.18% and 96.56%, respectively. The resistance frequencies for the rice blast and the length of lesions resulting from BB ranged from 96.7% to 100% and 0.77 to 1.18 cm, respectively. A new cytoplasmic male sterile line, Rongfeng 3A, with *Pi1*, *Pi2* and *Xa23*, was successfully developed through successive backcross breeding. MABC has been successfully utilized for developing elite parental lines of hybrid rice with inbuilt resistance to BB and blast diseases.[69–71] The hybrid Pusa RH10 is more susceptible to BB and blast. Pusa RH10 would improve its adaptability to disease endemic region and also sustain the productivity of the rice by incorporating the bacterial and blast resistance gene. In order to detect QTLs controlling blast resistance, mapping population of 192 F_2:3_ families derived from the cross of two Iranian rice varieties Tarom Mahali (TAM), blast susceptible cultivar and the blast resistance cultivar cul, Khazar (KHZ) were developed. By using 74 polymorphic markers, two parental lines TAM and KHZ showed high genetic variability between the two varieties on chromosomes 1, 2, 3, 4, 5, 9, 11 and 10.[72]

#### Bacterial blight resistance

A novel BB resistance gene, *Xa23*, identified from *Oryza rufipogon* was introgressed into three popular restorer lines (Minghui63, YR293 and Y1671) for wild abortive cytoplasmic male sterility by marker-assisted backcross breeding approach in combination with artificial inoculation and stringent phenotypic selections. Foreground selection against *Xa23* gene was used to select plants carrying *Xa23* gene prior to transplanting through MAS using a closely linked SSR marker (RM206) of 1.9 cm apart from the *Xa23* locus. [73] The newly developed BB resistant restorers and their derived hybrids were identical to their respective original versions for agronomic traits especially under disease free condition.[71] In the Philippines, the gene combination of *Xa4*+*Xa5*+*Xa21* derived from two rice cultivars (NSIC Rc142 and NSIC Rc154) for blight resistance have been integrated into the susceptible cultivar IR64 genetic background using MABC.[35] Recently, it has been successfully incorporated two genes for BB resistance namely, *X* *a13* and *Xa21* in the restorer line PRR78 using MABC.[70]

In lowland rice in Thailand, BB resistance gene *Xa21* obtained from rice variety IR1188 was introgressed into popular variety KDML105 through three rounds of MABC and phenotypic selection. The developed introgression lines carrying BB resistant gene (*Xa21*) have showed the characteristics identical to the local variety KDML105 resulted from different locational tests at research centre in lowland rice cultivation area.[29] In their study, three seedling resistance loci inherited from IR1188 were identified on rice chromosomes 1 (RM302, RM212), 8 (RM210, RM149) and 11 (RM287, RM224). Sixty-seven rice microsatellite marker (SSR) well distributed over the whole rice genome, together with markers that are associated with the cooking characteristics of KDML105, were used to determine the background genotype of the Koshihikari backcross inbred lines (KBILs).

In China, BB resistance genes *Xa7* and *Xa21* derived from Huahui20 have been introgressed into the popular restorer line, Yihui1577 using the MABC approach.[30] The genotype of each BC progenies was determined using tightly linked SSR marker, RM20582 in the presence of *Xa7* to map a 0.14 cM interval between the markers RM20582 and RM20593 on chromosome 6.[74] At BC_3_F_1_ generation, plants were selfed to produce BC_3_F_2_ population. Homozygous plants carrying *Xa7*, *Xa21* and both of them were selected in BC_3_F_2_ to produce BC_3_F_3_ families. In China, a successful introgression of four BB resistance genes (*Xa7, Xa21, Xa22* and *Xa23*) derived from cytoplasmic male sterile line Jinke 1A, was done into an elite hybrid rice restorer line Huahui 1035 through MABC.[31] Result revealed that three promising high-yielding F_1_ hybrid lines were selected for hybrid rice production in China. Two BB resistance genes *Xa21* and *Xa13* obtained from an elite high-yielding, fine grain-type variety, Samba Mashuri has been introgressed into BB susceptible two elite varieties, Taraori Basmati and Basmati 386 by using the MABC programme.[27]

#### Gall midge resistance

Rice gall midge resistance gene *Gm8* achieved from donor parent Aganni, has been introgressed into an improved variety Samba Mashuri carrying gene *Xa21* through MABC. Results revealed that in BC_2_F_2_, four plants were tested and Gm8 and *Xa21* genes were identified. In F_3_ progeny, these plants were phenotyped for resistance to BB and gall midge.[53] Breeding for gall midge resistance has been one of the most successful stories of modern crop improvement. However, rapid evolution of virulent biotypes against the resistant rice varieties carrying a single major gene during the 1980s and thereafter [75] has called for a rethink of the breeding approach. So far, eleven resistance genes in the plant [76] and seven biotypes of the pest have been reported.[77]

#### Virus resistance

An RNA interference construct (ORF IV of RTBV, placed both in sense and anti-sense orientation under CaMV 35S promoter) was transferred to two high-yielding Tungro-susceptible *indica* rice cultivars (IET4094 and IET4786) from the transgenic Pusa Basmati (PB-1) scented rice line using backcross breeding till the BC_2_F_3_ stage and the progenies (BC_2_F_1_) showed mild symptoms of Tungro, in contrast to severe symptoms displayed by the RPs.[78] In another research, two QTLs were derived from an upland resistant *japonica* variety ‘Azucena’ which were partial resistance to *Rice* *yellow mottle virus* (RYMV) (located on chromosome 12 and 7) have been introgressed into a lowland susceptible *indica* variety ‘IR64’ using MABC.[79] In their study, the valuation of RYMV was done in F_2_ and F_3_ progenies of BC_1_ and BC_2_ generations. In BC_3_ progeny, it was the highest recovery of the RP genome i.e. 95% for the 10 non-carrier chromosomes.

#### Brown plant hopper resistance

Brown plant hopper resistant gene *bph3* derived from donor cultivar ‘Rathu Heenati 'has been transferred into the most popular Thai rice variety 'Khao Dawk Mali 105’ (KDML105).[80] In this scheme, two SSR markers, RM589 and RM190, closely linked to Bph3 and Wx-RH loci, respectively, were used to identify the genotype of BC_3_F_2_ individuals. Only progenies carrying heterozygous genotypes at the Wx-RH and Bph3 region were selected to generate BC_3_F_3_. A total of 75 polymorphic SSR markers distributed throughout rice genome were then used to determine the recurrent genetic background of the 50 selected ILs from the BC_3_F_4_ generation.

Recently the *Bph18* gene from donor *indica* line IR65482-7-216-1-2 has been successfully transferred to Junambyeo, an elite *japonica* cultivar, by MAS and several generations of backcrossing.[52] The most tightly linked co-dominant sequence-tagged site (STS) marker, 7312T4A was used to detect the presence of the *Bph18* gene in backcross-derived BPH-resistant breeding lines.[81] Some 260 SSR markers of known chromosomal positions distributed evenly on the 12 chromosomes with an average interval of 5.9cM were used in a genome-wide survey to identify the chromosome segment substitution locations in the four advanced backcross breeding lines compared with the donor line. The SSR markers polymorphic between the two parents were used for background genotyping to recover the recipient parent genome.

### Marker-assisted backcrossing in abiotic stresses

#### Submergence tolerance

Submergence s*ub1* gene at the locus RM23805 derived from IR64 was incorporated into susceptible variety OM1490. The band corresponds to an allele from susceptible parent OM1490 and tolerant one IR64-Sub1 as 240 and 230 bp bands, respectively, at the locus RM23805.[44] *sub1A* gene has been introgressed into a popular high-yielding variety from India, Swarna following MABC procedure within 2 years.[12] In Vietnam, successful introgression *sub1* derived from donor rice variety IR64, has been done into popular rice variety AS996 through MABC. The introgression of *sub1* was confirmed using ART5 and SC3.[43] Parental diversity was carried out with 460 markers, 53 polymorphic markers of which were used for assessment on BC_1_F_1_, BC_2_F_1_ and BC_3_F_1_ generations having recipient allele 87.5%, 93.75% and 96.15%, respectively. In BC_4_F_1_, there was the highest genetic background i.e. 100%. All mega varieties (Samba Mahsuri and CR1009 from India, IR64 from the Philippines (IRRI), Thadokkham 1 (TDK1) from Laos and BR11 from Bangladesh with *sub1* introgression had a significantly higher survival rate than the original parents by using the MABC strategy. The tolerance level of an intolerant *sub1C* allele combined with the tolerant *sub1A*-1 allele did not significantly reduce and the expression of *sub1C*-1 was independent over the *sub1A* allele. Plants remained intolerant when *sub1C*-1 expression was completely turned off in the presence of *sub1A*-2. Survival rates and *sub1A* expression were significantly lower in heterozygotes compared with the homozygous tolerant parent.[82]

#### Salt tolerance

Very recently in Vietnam, the *Saltol* QTL obtained from the highly salt tolerant rice variety FL478 has been transferred into the high-yielding and widely grown cultivars, ASS996 by following the MABC strategy.[47] In this study, in each backcross generations, AP3206, RM3412 and RM10793 were used for screening heterozygous plants and 63 polymorphic markers were used to be distributed on 12 chromosomes. Two plants P284 and P307 had the highest recipient alleles up to 89.06% and 86.36% were used to develop BC2F1 populations. The recombinant selection was done with RM10694, RM562, RM7075 along the *Saltol* region on chromosome 1. Plant P284-112-209 was the best BC_3_F_1_ individual with all the recipient alleles screened based on a total of 63 markers. The four plants P307-305-21, P284-112- 195, P284-112-198 and P284-112-213 were the second ranking with only one loci heterozygous. All those five plants were chosen as the breeding lines for result of *Saltol*-AS996 introgression.

*Saltol* QTL derived from FL478 was introgressed in genetic background of Bacthom 7 cultivar. The background analysis in the introgression line revealed the recovery up to 96.8%–100% of RP alleles based on the screened markers after three generations.[48] In this study, 8 markers were used to identify *Salto*l locus and 81 markers were used in other loci between the parents. Then, 88 markers were applied to analyse genotyping of each backcross generation with the three steps of selection (foreground, recombinant and background). The results revealed that the best plant of BC_3_F_1_ generation bear the highest recovery of recipient genome i.e. 96.8%–100%. This study revealed that the introgression lines can be directly developed into the salinity tolerance variety, which is suitable for cultivating in coastal areas of the Vietnamese Deltas using MABC.

#### Drought tolerance

Near isogenic lines (NILs) were developed by introgression of three root QTLs from CT9993, an upland *japonica* into IR20, a lowland *indica* cultivar through MABC procedure.[49] Among the NILs considerable, variation in drought response and grain yield under rain-fed condition in target populations was monitored. Among the 41 NILs, only 5 showed high yield permanence in both rain-fed and irrigated conditions compared to the IR20. Two NILs namely, 212 and 297, with three and two root QTLs, respectively had thicker and longer nodal roots and higher total and deep nodal roots weight than IR20. In addition, NIL 297 had higher nodal root volume and surface area, while NIL 212 had increased number of nodal roots compared to IR20. QTLs for drought tolerant traits were transferred into Thai variety KMDL105 by following MABC programme. The backcrossing with target selection resulted in 103 KMDL105 introgression lines carrying 1, 2 or 3 target combinations, where 79, 20 and 4 lines were derived from KDML105 x IR68586-F2-CA-143 (DH212) (cross 1), KDML105 x IR68586-F2-CA-31 (DH103) (cross 2) and KDML105 x IR68586-F2-CA-54 (DH126) (cross 3) crosses, respectively. Genome scanning revealed that carrier chromosomes in all crosses showed a low percentage of the recipient parent genome. Also, non-carrier chromosomes, especially in crosses 1 and 2, were found to carry segments of the donor, which was reflected in the low percentage of the recipient genome of KDML105 in all crosses.[83]

#### Phosphorus tolerance

*Pup1* has been introgressed into two irrigated rice varieties and three Indonesian upland varieties by using MABC approach. The first assessment of phenotypic of introgression lines suggests that *Pup1* is effective in different genetic backgrounds and environments and it has the potential to increase significantly the grain yield under field conditions.[50]

#### Grain fragrance

A molecular marker for grain aroma was first observed by Ahn et al [84]. The genomic clone RG28, a restriction fragment length polymorphism (RFLP) marker, was tightly linked to the fragrance locus in rice chromosome 8 at a genetic interval of 4.5 cM.[85] RG28 can be changed by sequencing into a STS marker and utilized as a marker for the polymerase chain reaction (PCR) of genomic DNA from rice varieties differing in their aroma.[86] Using this locus, different PCR-based markers were developed that can discriminate between aromatic and non-aromatic rice cultivars.[87,88] MABC has been successfully used for the improvement of aromatic rice. The quality traits of the Manawthukha cultivar were promoted by MABC, using Basmati 370 as a donor [89,90] suggested the progress of improved Pusa Basmati 1 and the improved versions of PRR78 successfully utilizing MABC.

#### Semi-dwarfing

The rice semi-dwarfing gene, *sd1*, has been studied intensively due to its contribution to the increase of crop production. Although *sd1* breeding was extensively applied since the 1960s, the recent advances in the molecular basis of this gene allowed designing a more precise breeding strategy – MABC – to track *sd1* introgression in two traditional rice varieties. MABC and background selection revealed as useful tools to assist breeding for semi-dwarfism in traditional rice varieties (*japonica*).[91]

### Cost effective of MABC programme

There are many factors like inheritance of the trait, method of phenotypic evaluation, field/ glasshouse and labour costs, and the cost of resources influence the cost of utilizing MABC. In some case, MABC is not much cheaper than phenotypic screening.[92,93] But in other cases, phenotypic evaluation is more time consuming and difficult. Using markers, may be cheaper and preferable.[93–95] From the simulation studies, in some cases, MABC has the ability to improve selection efficiency over phenotypic selection in breeding programmes.[21,96–98] Factors that influence the cost of utilizing MABC or MAS include inheritance of the trait, method of phenotypic evaluation, field/glasshouse and labour costs, and the cost of resources. By using DNA markers, the evaluation of backcross progenies may be expensive. Before starting MABC programme, an approximate cost estimate should be calculated. To minimize the costs, the protocols of marker genotyping should also be standardized and optimized. Initially the cost of MABC would be more expensive compared to the conventional breeding in short-term, but it would be economically benefitted by saving time. This conception is very much an important consideration for our plant breeders, because the accelerated release of an improved variety may translate into more rapid profits by the release of new cultivars as soon as possible.[99] Estimates of cost including consumable and labor per data point for rice marker genotyping during MAS is US$ 0.30 to 1.00.[19]

## Conclusion and future prospects

With newer technologies and through advances in the field of genomics, the challenge for plant breeders is to judiciously utilize these novel tools in molecular marker-assisted breeding for developing commercially viable improved cultivars which address specific problems in different crops. This can be made possible only when MAS is integrated into traditional breeding practices, rather than being considered as a substitute. Plant breeding keeps a great contribution in the rice production and balancing the food security year after year. It is a fact that rice researchers faced many difficulties for doing their research due to global warming and climate change. As a result, various types of new diseases and insects, and several biotic and abiotic stresses occur, which often decreases the rice yield. In this aspect, advances in rice biotechnology and genomics have paved the way to meeting the challenges and new genes for resistance to biotic and abiotic stress and MABC has already proven to transfer major genes into elite rice parents, using both foreground and background selection. Now MABC is more popular to rice researchers as a potential and simple technique, because the major benefit of the MABC technique is to use the varieties that are already well accepted by farmers so that the improved variety will be ensured of possessing the target traits prized by the farmers. In rice, the existence of popular varieties that are already grown offers the opportunity to use this approach with new traits. In addition, the MABC approach plays vital role for basic research applications in rice to develop new varieties with much greater precision than conventional backcrossing.

MABC has generated a good deal of expectations, which in some cases led to over-optimism and in others to disappointment, because many of the expectations have not yet been realized. Although there is the consideration of bright future possibilities and potentials effects of MABC, there are also some constrains to its use, including equipment, infrastructure, skilled manpower, poor private sector involvement, supplies or consumables. If we consider the financial support in agricultural sector in many developing countries, their development priorities are not included for genetic enrichment programmes using molecular tools. Various stages in the MABC development and application process were regarded as being costly. The most significant cost prior to MABC is the development of a genetic linkage map for the species of interest and identification of associations between genes or QTLs and economically important traits. Such cost could be significantly high for developing countries. In developing countries, in order to take up breeders, the returns to investments should be far superior compared with those in developed countries, given the significant opportunity costs and various constraints associated with availability of facilities and supplies. We expect that the cost of MABC can be decreased, resources pooled and shared and a capacity to be developed if we make good partnership between developing and developed countries, including public–private sector collaboration. Currently, the cost of utilizing markers is possibly the most important factor that limits the implementation of MABC. Therefore, new marker technology can potentially reduce the cost of MAS considerably. If the effectiveness of the new methods is validated and the equipment can be easily obtained, this should allow MABC to become more widely applicable for crop breeding programmes, especially in rice. An efficient cost-effective MABC technology must be developed that will allow breeders to assess the genotype across the full genome and to recombine genes of agronomic importance from diverse sources.
